# Production of soluble regulatory hydrogenase from *Ralstonia eutropha* in *Escherichia coli* using a fed-batch-based autoinduction system

**DOI:** 10.1186/s12934-021-01690-4

**Published:** 2021-10-18

**Authors:** Qin Fan, Peter Neubauer, Matthias Gimpel

**Affiliations:** Chair of Bioprocess Engineering, Technische Universität Berlin, Straße des 17. Juni 135, 10623 Berlin, Germany

**Keywords:** Regulatory hydrogenase, Difficult-to-express protein, *Ralstonia eutropha*, *Escherichia coli*, IPTG autoinduction, Lactose autoinduction, Fed-batch like cultivation, EnPresso B

## Abstract

**Background:**

Autoinduction systems can regulate protein production in *Escherichia coli* without the need to monitor cell growth or add inducer at the proper time following culture growth. Compared to classical IPTG induction, autoinduction provides a simple and fast way to obtain high protein yields. In the present study, we report on the optimization process for the enhanced heterologous production of the *Ralstonia eutropha* regulatory hydrogenase (RH) in *E. coli* using autoinduction*.* These autoinduction methods were combined with the EnPresso B fed-batch like growth system, which applies slow in situ enzymatic glucose release from a polymer to control cell growth and protein synthesis rate.

**Results:**

We were able to produce 125 mg L^−1^ RH corresponding to a productivity averaged over the whole process time of 3 mg (L h)^−1^ in shake flasks using classic single-shot IPTG induction. IPTG autoinduction resulted in a comparable volumetric RH yield of 112 mg L^−1^ and due to the shorter overall process time in a 1.6-fold higher productivity of 5 mg (L h)^−1^. In contrast, lactose autoinduction increased the volumetric yield more than 2.5-fold and the space time yield fourfold reaching 280 mg L^−1^ and 11.5 mg (L h)^−1^, respectively. Furthermore, repeated addition of booster increased RH production to 370 mg L^−1^, which to our knowledge is the highest RH concentration produced in *E. coli* to date.

**Conclusions:**

The findings of this study confirm the general feasibility of the developed fed-batch based autoinduction system and provide an alternative to conventional induction systems for efficient recombinant protein production. We believe that the fed-batch based autoinduction system developed herein will favor the heterologous production of larger quantities of difficult-to-express complex enzymes to enable economical production of these kinds of proteins.

**Supplementary Information:**

The online version contains supplementary material available at 10.1186/s12934-021-01690-4.

## Background

Recombinant protein production in *Escherichia coli* is most extensively performed under the control of lactose-inducible promoter systems [1]. Alternative to manual IPTG induction of gene expression from these P_*lac*_-derived promoters, autoinduction is a simple method for induction of recombinant protein synthesis. Cultures can be simply inoculated into autoinducing medium and grown to saturation without the need to follow the growth of the culture and add the inducer at an appropriate time. This provides huge advantages in high-throughput applications and time scheduling of shake-flask experiments. Lactose can be used as the potent and cheap replacement of IPTG as inducer for recombinant protein synthesis [2–6]. In principle, the *lac* operon is induced when allolactose derived from lactose by the active intracellular β-galactosidase, binds and inactivates the LacI repressor, thereby as a consequence of lactose uptake into the cells [7]. Autoinduction is based on the glucose catabolite repression and inducer exclusion phenomenon which is initiated by the presence of glucose [8–11]. When carbon substrate mixtures of glucose, lactose or glycerol are used, glucose is preferentially consumed followed by glycerol and lactose that also acts as inducer of P_lac_ controlled genes [12]. Studier and colleagues have explored and optimized autoinduction media. They have been successfully applied for production of target proteins in small and large scale using T7 expression systems [5, 13–15]. Interestingly, these lactose autoinduction media provided higher cell densities and target protein yields compared to manual IPTG induction in commonly used media [5, 16, 17]. Unlike IPTG, lactose is taken up by the *lacY*-encoded lactose permease and metabolized to the actual inducer allolactose in *E. coli*, resulting in a delayed induction effect [7]. Moreover, the induction effect by lactose is highly sensitive to the glucose concentration as catabolite repression results in reduced lactose uptake rates at excess glucose [7, 18, 19]. Furthermore, several defined autoinduction media including various amino acid and vitamin supplements have been successfully applied to label target proteins with isotopes or selenomethionine [5, 20–23]. Generally, glycerol as a growth-supporting carbon and energy source in lactose autoinduction media supports high cell density [24, 25]. However, in such system, recombinant protein production is highly dependent on the oxygenation level, particularly reduced production at high aeration rates due to an altered order of carbon source preference from lactose to glycerol has been observed [12]. This strong oxygen-sensitivity of protein production complicates scalability and reproducibility of autoinduction cultures or restricts the protein yields at low oxygenation level due to lower cell densities [26, 27].

Recently, the use of a combination of lactose and glucose-limited EnPressoB fed-batch medium demonstrated that glucose limitation rather than glucose starvation is important for *E. coli*, consequently protein expression works well with a continuous biocatalytic glucose release from dextrins [28–31]. Unlike in glycerol-based autoinduction media, in EnPresso-based medium lactose only serves as inducer but is not catabolized as carbon source thereby reducing the oxygen sensitivity of lactose autoinduction [31, 32]. The slow glucose release does not prevent lactose autoinduction and the lactose concentration stays approximately constant at reasonable high cell densities (OD_600_ > 20) during 24 h cultivation [32].

[NiFe]-hydrogenases are generally heterogeneous, multicomplex, metalloenzymes [33, 34]. According to their physiological function, composition and localization in the organism, [NiFe]-hydrogenases have so far been classified into five distinct groups, including membrane-bound uptake hydrogenases (MBH), cytoplasmic uptake hydrogenases and H_2_ sensors (cyanobacterial, H_2_-sensing hydrogenases), bidirectional hydrogenases containing additional subunits for binding soluble substrates (NAD^+^ and/or NADP^+^, MV, F420), membrane associated H_2_-evolving hydrogenases and high affinity H_2_-oxidizing hydrogenases [35, 36]. However, the functional core of the [NiFe]-hydrogenases is basically a heterodimeric protein with a large and a small subunit, although additional subunits are present in many of these enzymes [35, 37]. Interestingly, a few [NiFe]-hydrogenases are tolerant to O_2_ which is a prerequisite for their industrial application [35, 38, 39]. The O_2_-tolerant regulatory hydrogenase (RH) from *Ralstonia eutropha* H16 which is composed of the large subunit HoxC and the small subunit HoxB, together with the histidine kinase HoxJ they form the H_2_-sensing complex (HoxBC)_2_HoxJ_4_ [40–42]. More recently, the single HoxBC heterodimer isolated from *R. eutropha* has been shown to maintain its catalytic activity and its tertiary structure similar to the classic hydrogenases and was widely used in a number of in vitro spectroscopic studies [41, 43–47]. From structural and functional points of view, RH is superior to other hydrogenases and considered as valuable and simplest model system for studying multicomplex hydrogenases.

We have recently reconstituted the synthesis of the RH subunits HoxBC from *R. eutropha* in *E. coli* BL21 Gold under control of a P_lac_-derived promoter [48]. Using classical IPTG induction soluble RH was produced in batch cultivations at a modest yield of 14 mg L^−1^. When switching to EnPresso-based fed-batch like shake-flask cultures the yield was increased more than 18-fold to approx. 255 mg L^−1^ [48], corresponding to several 100-folds increase in the RH amount purified from *R. eutropha* [44]. In this study, we aimed to examine the general applicability of autoinduction in combination with the EnPresso B glucose release growth system and the potential advantages of such a combined approach for the improvement of recombinant protein production compared to the classical single-shot IPTG induction. Using screening experiments in deepwell plates we investigated optimal IPTG and lactose concentrations for autoinduced RH production under fed-batch like conditions. Compared to classical IPTG induction about twofold higher volumetric yields and even fourfold higher space–time yields were obtained with lactose autoinduction. Finally, the heterologous RH production in *E. coli* achieved using fed-batch based lactose autoinduction showed a good scalability in shake flask scales and can be applied to rationally designed bioreactor cultivations.

## Results

### Effect of concentrations of glucose/lactose ratio on RH production in EnPresso B-based autoinduction

**Fig. 1:**
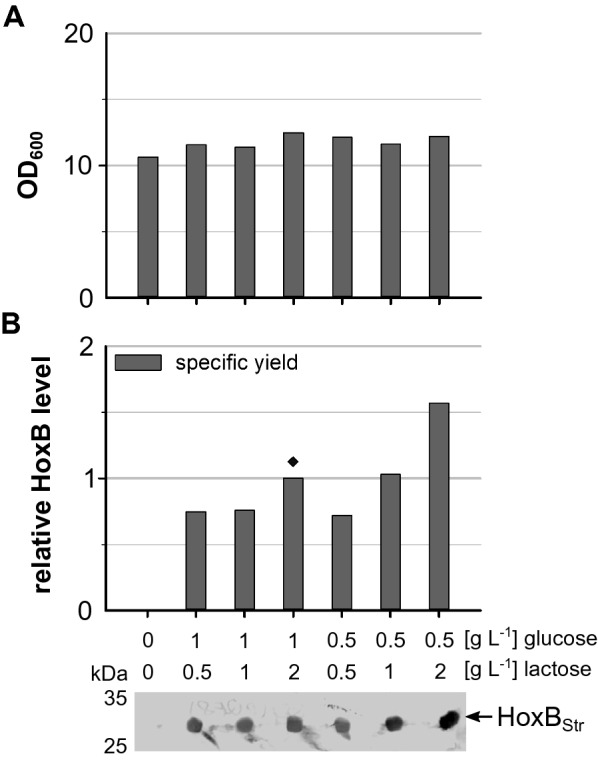
Effect of glucose and lactose on cell growth and HoxB production with autoinduction. *E. coli* BQF8RH was cultivated in 3 mL non-boosted EnPresso B medium on a 24-deepwell plate at 30 °C, 250 rpm for 24 h. Different concentrations of lactose and glucose were added as indicated to trigger RH production. **A** Final cell densities. **B** Samples normalized to OD_600_ of 5 were taken and HoxB detected by Western blotting. Specific HoxB yields were calculated relative to the HoxB yield reached in autoinduction with 1 g L^−1^ glucose and 2 g L^−1^ lactose (indicated by ♦)

Derivatives of the *lac* promoter are widely used in biotechnological processes for the controlled regulation of gene expression. Usually production of the recombinant protein is induced by a single shot addition of the artificial inducer IPTG at a certain point of the cultivation. In contrast, lactose-based autoinduction is a simple and rapid way to obtain high yields of a recombinant protein. In an autoinduction medium, the presence of glucose inhibits induction by lactose through catabolite repression and inducer exclusion [5]. To evaluate the suitability of autoinduction for heterologous production of the *R. eutropha* RH, we used *E. coli* BL21-Gold derivative BQF8RH (*E. coli* BL21-Gold transformed with plasmid pQF8 carrying the RH structural genes *hoxB* and *hoxC* under control of the P_lac-CTU_ promoter) that allows IPTG-inducible RH gene expression [48]. Cultures were performed in 24-deepwell plates (3 mL working scale) containing non-boosted EnPresso B medium supplemented with different amounts of glucose (0.5 or 1 g L^−1^) and lactose (0.5, 1 or 2 g L^−1^). Similar cell growth of the *E. coli* strain was observed with all glucose and lactose combinations added (Fig. 1A). The highest cell density with OD_600_ of 12.5 was reached with addition of 1 g L^−1^ glucose and 2 g L^−1^ lactose (Fig. 1A). Thus, despite the addition of higher amounts of glucose and lactose, no noticeable effect on growth was observable. The specific production of HoxB was detected by Western blotting. As expected, no hydrogenase was produced without addition of lactose, while HoxB_Strep_ was detectable in all other samples (Fig. 1B), indicating that lactose based autoinduction is feasible for RH production in *E. coli*. The highest RH level was observed in EnPresso supplemented with 0.5 g L^−1^ glucose and 2 g L^−1^ lactose.

### Screening of lactose concentrations for autoinduction of RH production

**Fig. 2:**
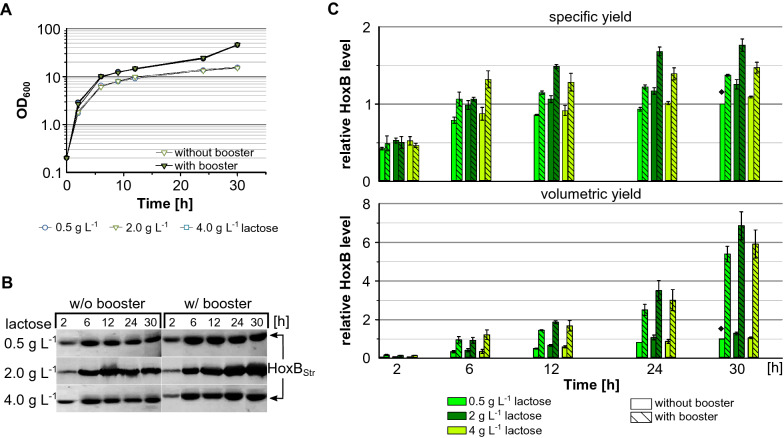
RH production with autoinduction by varying concentrations of lactose. *E. coli* BQF8RH was cultivated in 3 mL EnPresso B medium with/out booster on 24-deepwell plate at 30 °C, 250 rpm for 30 h. Autoinduction was achieved by addition of different lactose concentrations as indicated and 0.5 g L^−1^ glucose. Samples normalized to OD_600_ of 5 were taken at the indicated time points and used for detection of HoxB levels by Western blotting (WB) **A** growth curve, **B** WB for detection of HoxB yields, **C** specific HoxB yield (top) and volumetric HoxB yield (bottom). Both specific and volumetric yield were calculated relative to the HoxB yield reached after 30 h in non-boosted EnPresso induced with 0.5 g L^−1^ lactose (indicated by ♦)

To further investigate the role of lactose autoinduction we compared RH production induced by different concentrations of lactose (0.5, 2 or 4 g L^−1^) during growth in boosted and non-boosted EnPresso as above. Again 0.5 g L^−1^ glucose was added to the cultivations to inhibit protein expression at the beginning. As expected, the boosted cultures reached final OD_600_s of approx. 45 after 30 h which is about threefold higher compared to the non-boosted cultures (Fig. 2A). At indicated time points samples normalized to OD_600_ of 5 were taken and HoxB_Strep_ production analysed by Western blotting with Strep-tag specific antibodies. The specific HoxB level steadily increased over the cultivation time in all cultures indicating that lactose based autoinduction of RH expression is feasible (Fig. 2B, C). In both boosted and non-boosted cultures, approx. 80% of the maximum specific RH level was already reached after 6 h with all inducer concentrations tested. Interestingly, HoxB was already detectable within 2 h of cultivation, indicating that the initial glucose concentration (0.5 g L^−1^) is low enough to allow lactose to trigger RH production during the early stage of cultivation. Furthermore, our results show that 2 g L^−1^ lactose is optimal for autoinduction, as this resulted in a slightly higher RH level relative to 0.5 or 4 g L^−1^ lactose. In addition, cultures with booster added from the beginning of the cultivation show a 1.4-fold higher specific RH level compared to non-boosted cultures (Fig. 2B, C) which is in good agreement with the previously observed positive effect of the booster [48]. Nevertheless, due to the higher cell density addition of booster resulted in a 4.5-fold higher final volumetric HoxB yield.

### Screening of IPTG concentrations for autoinduction of RH production

**Fig. 3:**
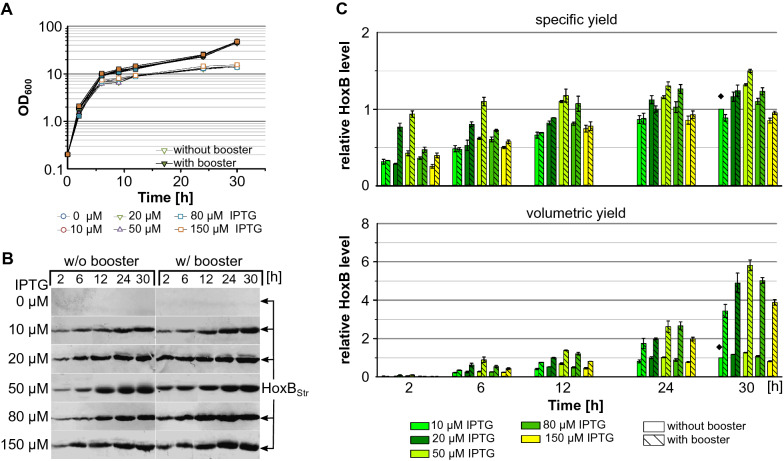
RH production with autoinduction by varying concentrations of IPTG. *E. coli* BQF8RH was cultivated in 3 mL EnPresso B medium with/out booster on 24-deepwell plate at 30 °C, 250 rpm for 30 h. Autoinduction was achieved by addition of different IPTG concentrations as indicated and 0.5 g L^−1^ glucose. Samples normalized to OD_600_ of 5 were taken at the indicated time points and used for detection of HoxB levels by Western blotting (WB). **A** Growth curve, **B** WB for detection of HoxB yields, **C** specific HoxB (top) and volumetric HoxB yield (bottom). Both specific and volumetric yield were calculated relative to the HoxB yield reached after 30 h in non-boosted EnPresso induced with 0.5 g L^−1^ lactose (indicated by ♦)

The conventional induction protocol for gene expression using P_lac_ derivatives is the single shot IPTG induction which is commonly performed after reaching a certain cell density. Recently, we have demonstrated that the single shot IPTG induction of the EnPresso B cultures with IPTG concentrations between 50 and 1000 µM provided fairly similar yields of recombinant RH protein whilst low IPTG concentrations proofed beneficial for the solubility of the recombinant protein [48]. To investigate the effect of IPTG autoinduction in EnPresse B medium on cell growth and RH production, we compared RH expression induced by different concentrations of IPTG (0, 10, 20, 50, 80 or 150 µM) performed in 24-deepwell plates. Analogous to autoinduction with lactose, 0.5 g L^−1^ glucose was supplied to the media to inhibit the IPTG uptake and subsequent *hox* gene expression at the beginning of the cultivation. As before, addition of booster facilitated cell growth and resulted in final OD_600_s above 40 which is threefold higher compared to the non-boosted cultures (Fig. 3A).

However, interestingly the strength of induction did not influence the growth neither boosted and nor for non-boosted cultures (Fig. 3A). HoxB was already detectable after 2 h of cultivation and the specific HoxB level steadily increased until 24 h of cultivation independent from booster and inducer concentration (Fig. 3C). Compared to induction with 10 or 150 µM IPTG, slightly higher RH titers were obtained by induction with 50 µM IPTG. Regardless of the inducer concentration, boosted cultures showed an approx. 1.5-fold higher HoxB level, which is in good accordance with previous results [48]. Again, the higher cell densities in boosted cultures resulted in about 5 to 6-fold higher volumetric yields compared to non-boosted cultures.

In summary, all cultures demonstrated a seemingly stable RH expression profile and steady RH accumulation during the whole cultivations. Here, the optimal concentrations for autoinduction seem to be 50 µM IPTG and 2 g L^−1^ lactose, respectively. Thus, autoinduction with lactose or IPTG can be used for recombinant RH production in fed-batch like EnPresso cultures.

### Effect of glucose polymer feeding on RH production

**Fig. 4:**
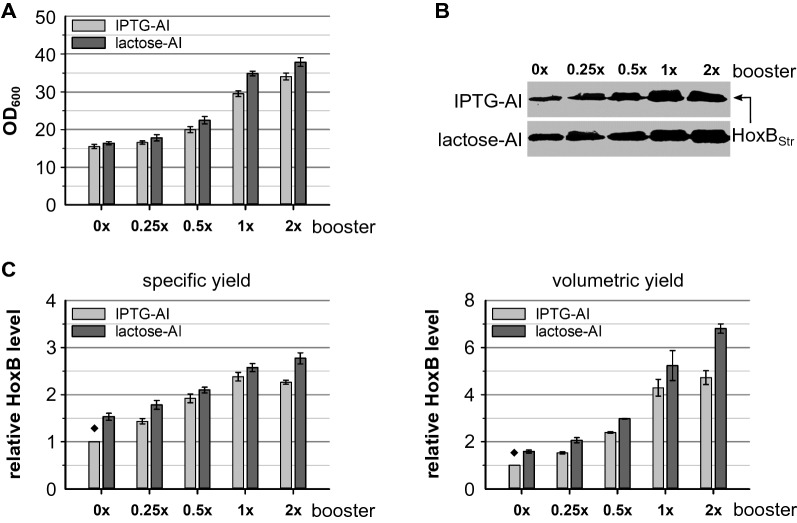
Effect of glucose polymer boosting on cell growth and RH production. *E. coli* BQF8RH was cultivated in 3 mL EnPresso B medium with varying booster concentrations (0 ×, 0.25 ×, 0.5 ×, 1 ×, 2×) on 24-deepwell plate at 30 °C, 250 rpm for 30 h. RH expression was autoinduced by 50 µM IPTG or 2 g L^−1^ lactose in the presence of 0.5 g L^−1^ glucose. Samples normalized to OD_600_ of 5 were taken at the end of cultivation and used for detection of HoxB levels by Western blotting (WB). **A** Cell growth, **B** WB for detection of HoxB yields, **C** specific HoxB yield and volumetric HoxB yield. Both specific and volumetric yield were calculated relative to the HoxB yield reached in 0 × boosted IPTG-autoinduced EnPresso culture (indicated by ♦)

Addition of glucose polymer to boost the EnPresso fed-batch like cultures improved final cell density and RH productivity in both classical IPTG induction [48] and autoinduction (see above). To further investigate the effect of the booster on RH production, we performed cultivation of *E. coli* BQF8RH in 24-DWPs with addition of different booster amounts. In all cases RH production was induced by either lactose or IPTG autoinduction. As expected, increasing amounts of booster gradually increased cell growth (Fig. 4A; Additional file 1: Fig. S1). Thus, this underlines the obvious influence of boosting concentrations on cell growth. Similarly, analysis of final RH levels using Western blotting (Fig. 4B) corroborates that RH production is positively affected by increased booster amounts. However, the highest specific yield is reached with 1 × booster and addition of 2 × booster did not significantly increase the specific RH yield further (Fig. 4C). Nevertheless, a slight increase in cell density with 2 × booster resulted in a slightly higher volumetric RH yield compared to the 1 × boosting. The same booster effect could be observed for both IPTG and lactose autoinduction. Moreover, comparable specific RH levels were observed independent from the autoinducer (Fig. 4B). Hence, lactose autoinduction according to the higher cell density (Fig. 4C) resulted in higher RH production titers which is consistent with the results described above.

### RH production with IPTG/lactose autoinduction in shake flasks

After initial screening trials in 24-deepwell plates the cultures were scaled up to a broth volume of 50 mL cultivated in 250 mL Ultra Yield shake flasks as described in “Materials and methods”. Protein production was induced for 24 h with 50 µM IPTG or 2 g L^−1^ lactose which were added into the medium together with 0.5 g L^−1^ glucose at the beginning of the cultivations. RH was purified by affinity chromatography and analysed by SDS-PAGE.

**Fig. 5:**
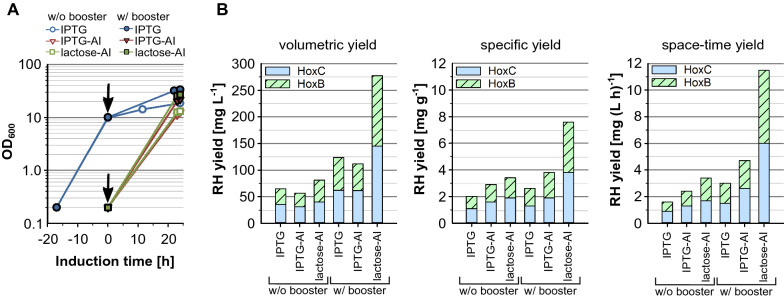
Soluble RH production in EnPresso B-based UYF shake flask cultivations. *E. coli* BQF8RH was cultivated in 50 mL EnPresso B medium with/out boosting in 250 mL UYF at 30 °C. For autoinduction 50 µM IPTG or 2 g L^−1^ lactose were added to induce RH production at the beginning of the cultivation. For comparison classic single shot IPTG induction was performed. For IPTG or lactose autoinduction cultures were induced from the beginning of the cultivation and cells harvested after 24 h of growth. For classic IPTG induction cells were cultivated for 18 h without inducer. Subsequently, RH production was induced by addition of 50 µM IPTG and finally cells were harvested after 24 h of induction resulting in a total process time of 42 h. The induction time points are indicated by black arrows. **A** Growth curve, **B** Specific, volumetric and space–time yield of RH calculated from Coomassie stained gels after affinity purification and quantification with ImageJ as described in “Materials and methods”

While the final OD_600_s after 24 h induction did not differ significantly between IPTG and lactose autoinduction cultures, compared to single shot induction with IPTG the final OD_600_ was about 1.5-fold lower (Fig. 5A). This is probably attributed to the significantly longer cultivation time when performing single shot induction, since induction is carried out after 18 h by which time the cultures have already reached an OD_600_ of about 10. Compared to classical single shot induction with IPTG, slightly lower volumetric yields were achieved with IPTG autoinduction, whereas specific yields were slightly higher (Fig. 5B). As a consequence of the shorter total process duration when using IPTG autoinduction, the space–time yield could be elevated about 1.6-fold.

By using 2 g L^−1^ lactose for autoinduction, the results of IPTG autoinduction could be significantly exceeded. Furthermore, higher volumetric yields were obtained both in EnPresso cultures with and without booster using lactose autoinduction compared to classical IPTG induction. In particular, in boosted EnPresso cultures, a final RH production titre of 278 mg L^−1^ could be achieved. The positive effect of lactose autoinduction on RH production is also reflected in the specific yield and the space–time yield of approx. 11.5 mg (L h)^−1^ in connection with the shorter process time, corresponding to fourfold increase compared to standard IPTG induction. As expected, for lactose and IPTG based autoinduction, booster addition improved the final volumetric and specific RH production by approximately 2- and 3-fold, respectively, compared to without booster. Therefore, it can be assumed that autoinduction provides an alternative way to enhance recombinant RH production yields.

### Enhanced RH production by repeated glucose polymer feeding

**Fig. 6:**
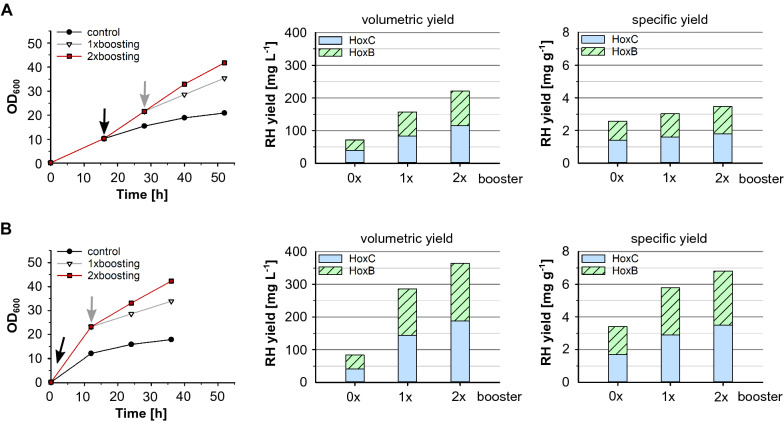
Glucose polymer boosting for soluble RH production. *E. coli* BQF8RH was cultivated in 50 mL EnPresso B medium with/without boosting in 250 mL UYF at 30 °C. Growth curves, specific and volumetric RH yield are presented. The RH yields were calculated from Coomassie stained gels after affinity purification and quantification with ImageJ as described in “Materials and methods”. **A** RH production using classic single-shot IPTG induction. **B** RH production using lactose-autoinduction. Black arrow: addition of booster and induction point; Grey arrow: addition of a second dose of booster

The continued glucose release by supply of additional polymer booster to the medium had a positive effect on cell growth and RH production as described above. Thus, we attempted to improve the RH production by repeated continued glucose supply. Parallel cultivations in shake flasks were carried out based on classic single-shot IPTG induction with 50 µM IPTG and lactose autoinduction using 2 g L^−1^ lactose (Fig. 6). The 1st dose of booster and biocatalyst was added at the induction point and a 2nd dose of booster and biocatalyst (4.5 U L^−1^ for IPTG induction, 3 U L^−1^ for lactose-autoinduction) was added after 12 h of induction. Without glucose polymer feeding, cells reached a final OD_600_ of 18–21 after 36 h of induction with IPTG or lactose, respectively. With glucose polymer feeding, the final cell density was remarkably increased in both cultivations up to an OD_600_ of 35 with single polymer feeding and even approx. 42 with repeated, two-times glucose polymer feeding. As before the addition of booster resulted not only in an increased biomass production but also in a higher specific RH yield. Consequently, the volumetric RH yield obtained with lactose autoinduction increased to 366 mg L^−1^. Overall, a nearly twofold increase in specific RH yields could be obtained with lactose autoinduction compared to IPTG induction with a maximum of 7 mg g^−1^ using lactose autoinduction with two-times glucose polymer feeding.

### Scalability of the RH production

To further evaluate the use of lactose autoinduction for RH production, cell densities and final RH yields obtained in deepwell plate and in 125 mL or 250 mL shake flask cultures were compared after 24 h of cultivation. In all three cultivation scales, comparable cell densities could be achieved regardless of the addition of the booster (Additional file 2: Table S1). However, cultivation in the 250 ml UYF resulted in slightly higher cell densities most likely due to the higher K_L_a values in the larger shake flask [49]. Similarly, increasing the size of the shake flask cultures had no significant negative effect on specific and volumetric RH yields (Additional file 2: Table S1). Thus, these results demonstrate the good scalability of the RH production process using fed-batch based lactose autoinduction over the shake flask and deepwell plate scales.

## Discussion

Recently, we demonstrated the functionality of the enzyme-based fed-batch like EnPresso system for the heterologous production of hydrogenases in *E. coli* using the regulatory hydrogenase from *R. eutropha* as a model [48]. With a classical IPTG induction, RH titers of 130–260 mg L^−1^ could be achieved in shake flask scale. Here, low IPTG concentrations of 50 µM favour the solubility of the RH and the use of the booster increases the specific as well as the volumetric yield [48]. Based on this, in the present work we wanted to evaluate the possibility of enhancing RH production using autoinduction. Initial experiments in deepwell plates indicated that heterologous RH production in *E. coli* using autoinduction with lactose is possible (Figs. 1 and 2). The comparison of different lactose amounts used for autoinduction indicated that an intermediate lactose concentration of 2 g L^−1^ is optimal for RH production. Even though 4 g L^−1^ lactose resulted in slightly higher final OD_600_, the highest specific and even volumetric RH yield was obtained after autoinduction with 2 g L^−1^ lactose (Fig. 2). This is in good accordance with the work of Ali [50] who proposed that low lactose concentrations (0.1—2.0 g L^−1^) would be more profitable for autoinduction in EnPresso B-based fed-batch like cultures, as high lactose concentrations (˃2 g L^−1^) would lead to a longer batch phase consequently resulting in more biomass but less protein production [50]. Likewise, the faster growth in the presence of high lactose concentrations could lead to oxygen limitation, which further reduces protein synthesis [50]. On the contrary, higher lactose concentrations have been reported to provide an additional benefit for better induction of prolonged protein expression in the autoinduction systems [51, 52]. However, the use of the EnBase fed-batch like system with slow gradual glucose feeding enables slow protein synthesis with slowly growing cells [53, 54]. Previously, 0.3 g L^−1^ have been calculated as the lowest external lactose concentration sufficient for induction of recombinant protein production in *E. coli* growing at the minimal growth rate, whereas 4.1 g L^−1^ lactose is essential to ensure induction at any growth rate [55]. The sufficiency of relatively low lactose concentrations for successful autoinduction in enzymatic glucose supply systems might be attributed to the constant lactose concentration observed during 24 h of cultivation [32]. In such systems, the continuous glucose release prevents an diauxic growth shift and consequently lactose consumption while the product is slowly but steadily produced in a correctly folded form [32]. In our case, 2 g L^−1^ lactose seems to be optimal for autoinduction of soluble RH production in EnPresso B fed-batch like cultured *E. coli*. Nevertheless, the minimal lactose concentration of 0.5 g L^−1^ was enough for induction of recombinant RH synthesis which is perfectly supported by the result of Mayer et al*.,* who proposed that slow growth might contribute to reduce the need of lactose [32].

Recently, it had been reported that IPTG can replace lactose in the classic lactose-based autoinduction system to enhance protein production in batch cultures [52]. At low IPTG concentrations (< 100 µM) the inducer is actively transported into the cell by the lactose permease LacY, while at higher IPTG concentrations used for normal induction (200–1000 µM) the inducer enters the cell by passive diffusion [52, 56]. Thus, the expression level is controlled by the IPTG uptake while the excessive inducer remains in the culture medium [57, 58]. Here, our results show that IPTG-based autoinduction is also suitable for heterologous production of RH. Although 10 µM IPTG was sufficient for the induction of RH expression, the highest yield was achieved with 50 µM IPTG (Fig. 3) which is in clear line with previous studies that only partial induction was achieved at IPTG concentrations below 40 µM. This is probably due to the fact that either the amount of IPTG is not sufficient to remove all repressor molecules from the operator site in the induced cells, or that only a subpopulation could be induced by the limited amount of externally available IPTG. [57, 58]. In our case, 50 µM IPTG seems to be enough for full induction of RH expression under control of the bistable *lac*-derived operon. RH production started already within the first 2 h of cultivation, indicating the absence of an induction delay which typically occurs in conventional glycerol-based autoinduction medium [12]. Our observations are in clear line with recent findings that protein synthesis occurs under conditions where the glucose concentration drops below 0.3 g L^−1^ in fed-batch like cultures [32]. Thus, we estimate that the initial glucose concentration of 0.5 g L^−1^ seems to be low enough to allow the uptake of IPTG or lactose and consequently inducing P_lac_-controlled gene expression in EnPresso-based cultures.

When the cultures were scaled up from squared deepwell plates to different UYF scales no significant difference in cell growth was observed. Despite 4 times higher K_L_a values in the 250 ml UYF compared to the deepwell plate [59, 60], similar final cell densities were achieved. This suggests that the availability of oxygen is not a limiting factor which is well supported by previous work showing a reduced sensitivity to the oxygenation level by lactose autoinduction [31]. Our results indicate that the process can be scaled up or down very well. Thus, the fed-batch based lactose autoinduction is suitable for automated high-throughput screenings as a starting point for the development of analogue bioprocesses aimed at the production of difficult to express proteins, e.g. hydrogenases. Additionally, the good scalability of the RH production process in the current shake flask scales (125 mL- and 250 mL-scale) the fed-batch based lactose autoinduction can be applied to rationally designed bioreactor cultivations. Recent studies have shown that the lactose autoinduction concept coupled with an enzymatic glucose release system, compared with glycerol-based systems, is more widely applicable to shaken and stirred bioreactor cultivations with different scales and different aeration capabilities [31, 32].

Compared to non-boosted EnPresso B cultures, booster supplementation increased the volumetric RH production by 2 to 3-fold, as glucose released from the booster provided an additional carbon source for cell growth and protein production resulting in higher protein yields. The importance of the booster is indicated by higher cell densities as well as higher specific RH yields (Fig. 4C) that could be reached with increasing booster concentrations. However, specific RH production did not significantly increase further when using 2 × booster (Figs. 4 and 6) indicating a crucial limit of the booster effect on the RH production capability of individual cells. Nevertheless, repeated boosting improved both specific and volumetric RH yield by 1.3-fold compared to single boosting and even by fourfold compared to non-boosted cultivation, which confirms the positive influence of the booster on RH production and is in good agreement with previous findings [61]. Here, our results highlight the high dependence of RH production yields on the cell densities under both classic single shot IPTG induction and autoinduction of EnPresso fed-batch like cultures.

A comparison of RH yields obtained from cultures in UYFs induced with either classical single shot IPTG induction or autoinduction further corroborated the use of autoinduction for hydrogenase production in *E. coli*. While no significant differences in the specific yield could be observed between both IPTG induction protocols, a fourfold increase in volumetric and specific RH yield was obtained by autoinduction with 2 g L^−1^ lactose compared to optimized single-shot IPTG induction, pointing out an alternative possibility of recombinant protein production in *E. coli*. Furthermore, the similar volumetric yields together with the shorter cultivation time resulted in a space time yield of nearly 12 mg (L h)^−1^ with lactose autoinduction in EnPresso B medium which is fourfold higher compared to standard IPTG induction.

Our results presented in this study reconfirm the applicability of such fed-batch lactose autoinduction and the use of IPTG in fed-batch like autoinduction for the production of recombinant proteins in general and of difficult-to-express proteins such as hydrogenases in particular. To date, despite extensive studies on autoinduction systems, all of them using optimized defined media have generally achieved higher final yields of target proteins by increasing final biomass compared to standard IPTG induction [14, 16, 47]. However, our fed-batch autoinduction system ultimately resulted in higher protein yields based on a similar biomass, probably due to an increased or maximized metabolic capacity of individual cell in favor of target protein production. Therefore, this interesting finding renders our developed system potentially advantageous over previous autoinduction systems and of practical implications in terms of improvement of recombinant protein production in the future.

## Conclusion

Here, we combined the autoinduction concept with the fed-batch like enzymatic glucose release technology to improve heterologous regulatory hydrogenase production in *E. coli*. Taken together, the findings of this study lay a foundation for using this developed approach for efficient production of other similar proteins in future studies of structural and functional characterization as well as biotechnological applications.

## Materials and methods

### Bacterial strain and culture medium

For all cultivations *E. coli* BQF8RH (BL21-Gold [*E. coli* B F^–^* ompT hsdS*(r_B_^–^ m_B_) dcm^+^ *Tet*^R^ *gal endA Hte*] harbouring plasmid pQF8) [48], was used. The plasmid allows production of both HoxC and C-terminally strep-tagged HoxB under control of the strong P_lac_ derivative P_lac-CTU_ [48]. If not stated otherwise all cultivations were carried out in EnPresso B medium according to the manufactures (EnPresso GmbH, Germany) and 25 µg mL^−1^ chloramphenicol were added.

### Cultivation in 24-deepwell plates

Screening of conditions for autoinduction of *hox* gene expression was performed in square-shaped 24-deepwell plates (24-DWP; Thomson Instrument Company) covered with sterile Breathable Film seal (Starlab, Hamburg, Germany) using EnPresso B with or without addition of booster in a working volume of 3 mL per well. In both cases 3 U L^−1^ biocatalyst were used to catalyse glucose release. In deviation from the manufacturer's instructions, the booster was added directly at inoculation. Cultures were inoculated to OD_600_ of 0.2 and incubated at 30 °C and 250 rpm with orbital shaking (25 mm offset, Infor HT, Switzerland). Cell density and target protein level were analysed at five time points during the cultivation (2, 6, 12, 24 and 30 h). For optimization of the inducer concentration varying concentrations of either IPTG (0, 10, 20, 50, 80 or 150 µM) or lactose (0.5, 2 or 4 g L^−1^) were added at the inoculation time. Additionally, different combinations of glucose (0.5 or 1 g L^−1^) and lactose (0.5, 1 or 2 g L^−1^) were used for autoinduction in non-boosted cultures.

Improvement of the booster concentration was performed in cultures induced with 50 µM IPTG or 2 g L^−1^ lactose. At the time of inoculation, different booster concentrations (0 ×, 0.25 ×, 0.5 ×, 1 ×, 2 ×) were added with 1 × booster corresponding to the manufacturer's recommendation (EnPresso GmbH, Germany). In all cases, glucose release was controlled by addition of 3 U L^−1^ biocatalyst at inoculation according to the manufacturer’s instructions. Cell density and target protein level were analysed after 30 h of cultivation. Optical densities (OD_600_) of the cultures were determined as described previously [48].

### Cultivation in shake-flasks

Cultivation in the shake-flask scale was carried out in 250-mL Ultra Yield Flasks (UYFs) sealed with a sterile porous membrane seal (AirOtop™) (Thomson Instrument Company) filled with 20% v/v EnPresso B medium with or without booster. For autoinduction, 50 µM IPTG or 2 g L^−1^ lactose were added. The shake-flask cultures supplemented with 0.01% v/v antifoam 204 (Sigma-Aldrich) were inoculated to OD_600_ of 0.2 and incubated at 30 °C and 250 rpm (25 mm offset, Infors HT, Switzerland). If required a second dose of booster and biocatalyst were added after 12 h of induction.

### Protein purification and SDS-PAGE analysis

Cells were harvested by centrifugation for 10 min at 8000 × *g* at 4 °C and pellets frozen at − 20 °C overnight. After thawing the pellets were resuspended in washing buffer A (100 mM Tris–HCl, pH 8.0, 150 mM NaCl) supplemented with 1 mg mL^−1^ lysozyme and 1 mM PMSF. Here, 4 mL of washing buffer were used per 1 g of wet cell weight. Subsequently, cells were disrupted by sonification (30 s on/off, sonotrode with 7 mm diameter, 60% amplitude) for 8–10 min. The resulting crude extract was centrifuged for 30 min at 16,000 × *g* at 4 °C to yielding the soluble protein extract. The clarified cell lysates were applied to *Strep*-Tactin® Gravity Superflow® columns (IBA GmbH, Germany) and processed according to the manufacturer’s instructions. For 10 mL of soluble extract, a 300 µL bed volume was used. The column was washed with 6 bed volumes of washing buffer, and the Hox proteins were eluted 6 times with 0.5 bed volume of elution buffer containing 2.5 mM D-desthiobiotin. Subsequently, elution fractions were analysed by SDS-PAGE using 12% PAA gels. BSA served as a standard for quantification. After staining with colloidal Coomassie solution the gels were scanned and subsequently bands quantified using Image J.

For total protein analysis 0.2 mL broth samples were collected at the selected time points, centrifuged at 16,000 × *g* and 4 °C for 10 min and pellets stored at − 20 °C. The pellets were resuspended in 2 × SDS sample buffer normalized to an OD_600_ of 5 and heated for 20 min at 95 °C. After centrifugation 20 µL of the SDS-denatured samples were loaded on 12% PAA gels as above.

### Western blotting

Western blotting was performed as described recently [48]. Briefly, after SDS-PAGE proteins were transferred onto a PVDF membrane (0.45 µm pore size, Carl Roth, Germany) by semi-dry blotting in a Transblot Turbo Transfer system (Bio-Rad, Germany) at 1.3 A/25 V for 30 min. Strep-tagged HoxB was detected using monoclonal anti-*Strep*-tag®II mouse antibody (1:2500 dilution) (iba GmbH, Germany). As a secondary antibody, alkaline phosphatase (AP)-conjugated goat anti-Mouse IgG antibody (Sigma-Aldrich, Germany) was used (1:8000 dilution). Bands were visualized via the AP-catalysed reaction of the chromogenic BCIP/NBT substrates as described recently [48]. Blots were digitized with a Scan-Prisa 640U and quantified with ImageJ.

## Supplementary Information

**Additional file 1: Figure S1.** Cell growth with different amounts of booster in IPTG and lactose autoinduction cultivations

**Additional file 2: Table S1.** Comparison of cell densities and volumetric RH production yields obtained from deepwell plate (DWP) to UltraYield flask (UYF) cultures (125 mL or 250 mL of flask volume) after 24 h of cultivation using lactose autoinduction.

## Data Availability

All data generated or analysed during this study are included in this published article [and its additional information files].
