# Supplementary material for: Production of soluble regulatory hydrogenase from Ralstonia eutropha in Escherichia coli using a fed-batch-based autoinduction system
Source: Microb Cell Fact. 2021 Oct 18;20:201. doi: 10.1186/s12934-021-01690-4 (PMC8522226; doi:10.1186/s12934-021-01690-4)
Supplement: Supplementary file 1 — Additional file 1: Figure S1. Cell growth with different amounts of booster in IPTG and lactose autoinduction cultivations [file 12934_2021_1690_MOESM1_ESM.pdf]

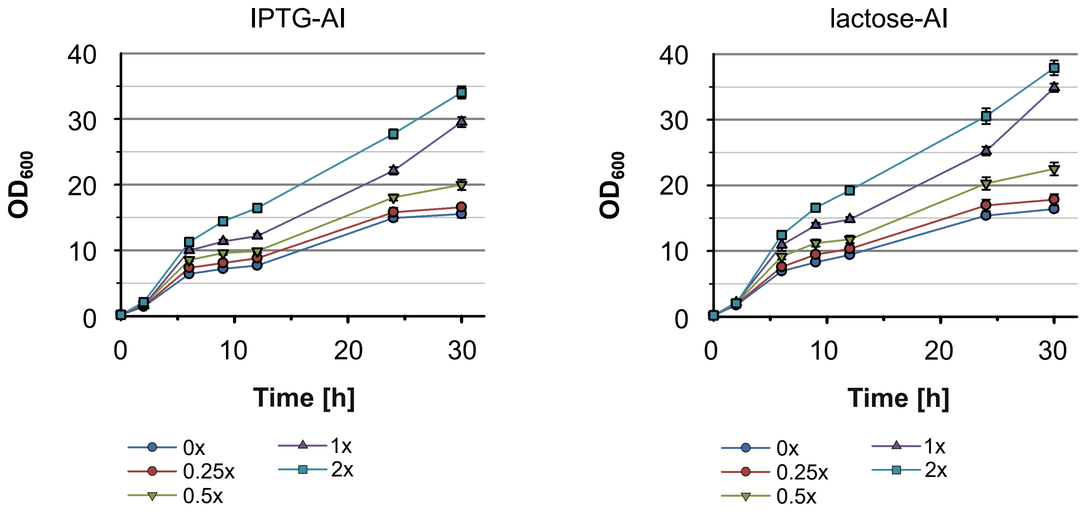

Figure S1 Cell growth with different amounts of booster in IPTG and lactose autoinduction cultivations. *E. coli* BQF8RH was cultivated in 3 mL EnPresso B medium with varying booster concentrations (0x, 0.25x, 0.5x, 1x, 2x) on 24-deepwell plate at 30°C, 250 rpm for 30 h. RH expression was autoinduced by 50 μM IPTG or 2 g L^{-1} lactose in the presence of 0.5 g L^{-1} glucose. OD_{600}s were measured at different time points.

Fan et al., Figure S1
