# Supplementary material for: Production of soluble regulatory hydrogenase from Ralstonia eutropha in Escherichia coli using a fed-batch-based autoinduction system
Source: Microb Cell Fact. 2021 Oct 18;20:201. doi: 10.1186/s12934-021-01690-4 (PMC8522226; doi:10.1186/s12934-021-01690-4)
Supplement: Supplementary file 2 — Additional file 2: Table S1. Comparison of cell densities and volumetric RH production yields obtained from deepwell plate (DWP) to UltraYield flask (UYF) cultures (125 mL or 250 mL of flask volume) after 24 h of cultivation using lactose autoinduction. [file 12934_2021_1690_MOESM2_ESM.pdf]

Table S1 Comparison of cell densities and volumetric RH production yields obtained from deepwell plate (DWP) to UltraYield flask (UYF) cultures (125 mL or 250 mL of flask volume) after 24 h of cultivation using lactose autoinduction.

**Culture conditions**		**OD_{600}**	**RH [mg L^{-1}]**	**RH [mg (L OD)$^{-1}$]**
non-booster	3 mL DWP	14.4	n.d	n.d
	125 mL UYF	13.8	80.3	5.8
	250 mL UYF	15.9	88.1	5.5
1 × booster	3 mL DWP	25.2	n.d	n.d
	125 mL UYF	21.9	131.7	6.0
	250 mL UYF	28.6	289.6	10.1
2 × booster	3 mL DWP	30.5	n.d	n.d
	125 mL UYF	23.8	243.1	10.2
	250 mL UYF	33.1	365.9	11.1

n.d: not determined

Fan et al., Table S1
